# P365: Management of biomedical waste of an Ivorian hospital

**DOI:** 10.1186/2047-2994-2-S1-P365

**Published:** 2013-06-20

**Authors:** N Yao, G Siogbo, EO Adjoumani-Gbohou, A Lokossué, AE Ablé, CS Kouassi

**Affiliations:** 1 Department of internal medicine HMA, Côte d'Ivoire; 2 National Training Institute for Health Agents, Abidjan, Côte d'Ivoire

## Introduction

Biomedical waste constitutes an environmental and public health problem. Inadequate management of those waste increases risks for health care professionals, for hospital patients and visitors and for the community.

## Objectives

The general objective of our survey was to assess the quality of biomedical waste management.

## Methods

The survey was conducted over a period of one month with health care staff members of Abidjan Military Hospital, which has been chosen adventitiously, and included questions on socio-professional characteristics, knowledge related to biomedical waste management and elimination modes. The expressed variables were in average (quantitative), in figures and percentage (qualitatives).

## Results

Forty people were surveyed, and the following findings were given: Most staff members were young people; 35% of them had a low education level; 60% of them were qualified. All surveyed staff recognized that biomedical wastes involve hazards. The best-known preventive actions included the use of security boxes and plastic pouches; 45.5% of them were not aware of appropriate waste management; 67.5% of them acknowledged that the screening is performed at the level of production; 25% did not perform washing nor disinfection of the equipment; 80% acknowledged that there was a storage area, and 75% acknowledged that there was a service responsible for collection and disposal; 75.6% of them acknowledged that the method used for disposal was the open pit burning.

## Conclusion

In Africa, the management of biomedical waste is inappropriately organized. To cope with that, it is important to train and raise awareness among healthcare staff. It is also important to have the appropriate equipment to process this waste.

## Disclosure of interest

None declared

